# Elevational Gradients in Fish Diversity in the Himalaya: Water Discharge Is the Key Driver of Distribution Patterns

**DOI:** 10.1371/journal.pone.0046237

**Published:** 2012-09-27

**Authors:** Jay P. Bhatt, Kumar Manish, Maharaj K. Pandit

**Affiliations:** 1 Centre for Interdisciplinary Studies of Mountain and Hill Environment, University of Delhi, Delhi, India; 2 Department of Environmental Studies, University of Delhi, Delhi, India; University of Massachusetts, United States of America

## Abstract

**Background:**

Studying diversity and distribution patterns of species along elevational gradients and understanding drivers behind these patterns is central to macroecology and conservation biology. A number of studies on biogeographic gradients are available for terrestrial ecosystems, but freshwater ecosystems remain largely neglected. In particular, we know very little about the species richness gradients and their drivers in the Himalaya, a global biodiversity hotspot.

**Methodology/Principal Findings:**

We collated taxonomic and distribution data of fish species from 16 freshwater Himalayan rivers and carried out empirical studies on environmental drivers and fish diversity and distribution in the Teesta river (Eastern Himalaya). We examined patterns of fish species richness along the Himalayan elevational gradients (50–3800 m) and sought to understand the drivers behind the emerging patterns. We used generalized linear models (GLM) and generalized additive models (GAM) to examine the richness patterns; GLM was used to investigate relationship between fish species richness and various environmental variables. Regression modelling involved stepwise procedures, including elimination of collinear variables, best model selection, based on the least Akaike’s information criterion (AIC) and the highest percentage of deviance explained (D^2^). This maiden study on the Himalayan fishes revealed that total and non-endemic fish species richness monotonously decrease with increasing elevation, while endemics peaked around mid elevations (700–1500 m). The best explanatory model (synthetic model) indicated that water discharge is the best predictor of fish species richness patterns in the Himalayan rivers.

**Conclusions/Significance:**

This study, carried out along one of the longest bioclimatic elevation gradients of the world, lends support to Rapoport’s elevational rule as opposed to mid domain effect hypothesis. We propose a species-discharge model and contradict species-area model in predicting fish species richness. We suggest that drivers of richness gradients in terrestrial and aquatic ecosystems are likely to be different. These studies are crucial in context of the impacts of unprecedented on-going river regulation on fish diversity and distribution in the Himalaya.

## Introduction

Understanding species richness patterns and factors influencing the species distribution is central to ecology and biogeography [1] and this information is critical to the conservation planning. Even as a number of hypotheses on the species richness patterns have been proposed by various researchers [2]–[4], there is lack of consensus regarding the factors influencing these patterns [5]. Studies on species richness patterns in different taxa along geographic gradients carried out so far have reported varying patterns [6]–[9] across geographic regions of the earth [3], [10], [11]. Species richness gradients are scale dependant and are driven by large-scale process (climate, resource availability and productivity), small-scale factors (habitat heterogeneity, disturbance, physiological tolerance, biotic interaction, dispersal limitation, etc.) and environmental variables that vary consistently along the geographic gradients [12], [13]. A framework for future studies on elevational diversity gradients outlined the need to identify the underlying drivers of species richness patterns [14]. The significance of such studies is linked to their importance in biodiversity conservation. In particular, when habitats are threatened by anthropogenic manipulations, understanding of species richness patterns is critical in facilitating the choice of species and habitats for conservation [15].

While numerous studies have been carried out on species richness patterns in plants [16], mammals [8], birds [9], not enough work is available on fishes (but see [17]). In particular, macroecological studies on fresh water fishes of mountain rivers are largely lacking (but see [18]). Upland freshwater rivers are considered particularly vulnerable to severe habitat changes due to river regulation for irrigation and electricity generation, followed by impending species losses [19], [20]. It is, therefore, crucial to develop scientific understanding of species rich regions such as biodiversity hotspots and montane ecosystems, which remain poorly explored biologically and are threatened by diverse challenges such as climate change and human manipulations for economic development [21], [22]. Considering the fact that all the three major river basins in the Himalaya – Sind (Indus), Ganga (Ganges) and Brahmaputra, and their tributaries are earmarked for nearly 300 new dam proposals, this study assumes greater relevance and significance (see [22], Grumbine RE and Pandit MK, unpublished results).

In this study we examine the relationship between elevation and fish species distributions in freshwater Himalayan rivers in the Indian subcontinent. To our knowledge, this is the first attempt to document fish species richness pattern for the entire Himalayan region and understand the drivers behind these patterns. We collated data from primary and secondary sources to examine the relationship between elevation, species richness, and species richness and environmental variables. With the help of analytical methods and modelling, we figured out the importance and influence of various environmental variables in driving fish species richness. Modelling has proven valuable in revealing biodiversity patterns [23]. The choice of variables used in the analysis largely depends on scale and type of study area and is also important for quality and the interpretability of models [24]. In particular, for aquatic ecosystems such as rivers, variables like water discharge, temperature and velocity, and physico-chemical parameters could play major role in influencing species richness especially in the fishes (see [17]).

In order to investigate which parameters control fish species richness in the Himalayan rivers, we quantified the richness of fish species in 16 Himalayan rivers as well as specifically in the Teesta river along with various sets of environmental variables across elevational gradients. Teesta river basin is a suitable study area for conducting ecological and taxonomic studies due to its wide elevational gradient extending from tropical to alpine ecosystems covered within a small geographical extent [25]–[29]. The modelling procedure involved use of regression techniques to illustrate species richness patterns and application of model predictors to forecast the most important driver of that pattern. We based our study on four different sets of variables: physico-chemical, biological, physiographic and topographic including a combination of the best fitting variables. These variables included water temperature, discharge and velocity, besides geographic area (surface area) of the drainage basin, drainage density, and biological factors (see [18], [30]).

Specifically, this study investigates the patterns of fish species richness in the Himalayan rivers and attempts to understand the factor/s and their level of influence in governing these patterns. In a wider sense, this contribution addresses three broad questions: (i) do species richness gradients in aquatic ecosystems follow same patterns as in terrestrial ecosystems? (ii) are the drivers of species richness same in terrestrial and aquatic ecosystems? and (iii) is there a case for realigning species-area theory to deal with aquatic ecosystems?

**Figure 1:**
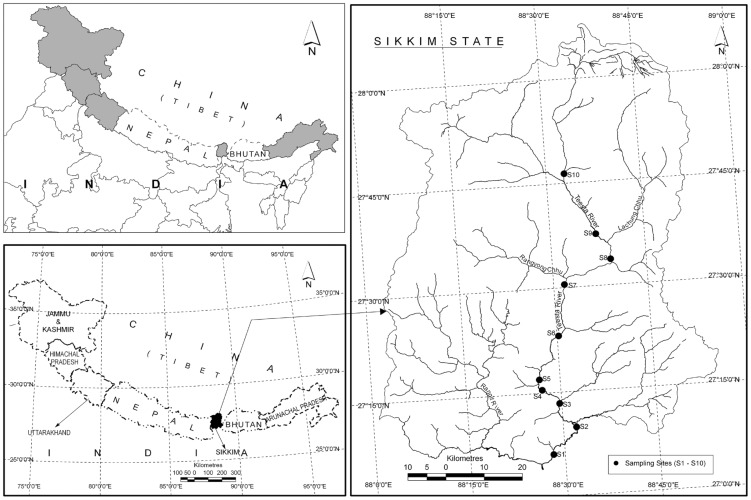
The location of the Himalaya and study area. Upper left: The Indian Himalaya (in grey shade), and Nepal and Bhutan Himalaya. Lower left: Geographical coordinates of the Himalaya (26°30′ –37° N latitude and 72°–97°30′ E longitude). Right: Geographical coordinates of Sikkim Himalaya and location of sampling sites along Teesta river that constituted our study area.

## Methods

### Study Area

Himalayan mountain ranges, located between 26°30′–37° N and 72°– 97°30′ E, stretch for about 2500 km between Nanga Parbat (8126 m) in the west and Namcha Barwa (7756 m) in the east, covering a geographic area of 594,400 km^2^ (Figure 1). The Himalaya is bounded in the west by the Hindu Kush and Karakoram ranges, in the north by the high Tibetan Plateau, in the south by the Plains of India and Pakistan and in the east and southeast by Bay of Bengal and Myanmar. The width of the Himalaya from south to north varies between 200 and 400 km at different places. The Himalayan basins are drained by 16 major rivers, which constitute three major river systems of the Indian subcontinent, namely Sind (Indus), Ganga (Ganges), and Brahmaputra. Though the river system continuum stretches for hundreds of miles, the fish zone in the Himalaya does not go beyond 4000 m elevation [31]. Water temperatures of the Himalayan rivers range between below 0°C in alpine areas to 22°C in glacier-fed rivers and 28°C in spring-fed rivers in the Himalayan foothills.

**Table 1:** Environmental variables considered for building of regression models of fish species richness in Teesta river.

Variable root	Description	Derivation	Model variables
**Physico-chemical set**			
Tu	Turbidity (ntu)	Primary sampling	Tu.avg
T	Water temperature (°C)		T.avg
TDS	Total dissolved solids (mg L^−1^)		TDS.avg
C	Electrical conductivity (µS cm^−1^)		C.avg
pH	pH		pH.avg
DO	Dissolved oxygen (mg L^−1^)		DO.avg
**Biological set**			
P	Phytoplankton (cells L^−1^)	Primary sampling	P.avg
B	Phytobenthos (cells cm^−2^)		B.avg
M	Macro-invertebrates (individuals m^−2^)		M.avg
**Physiographic set**			
D	River discharge (m^3^ s^−1^)	Primary sampling	D.avg
V	Water current velocity (m s^−1^)		V.avg
G	Gradient (m km^−1^)	Topographical map	G
**Topographic set**			
A	Basin area (km^2^)		A
S	Slope	DEM	MS, S, VS
	15–30% = moderately steep (MS),		
	30–50% = steep (S),		
	50–70% = very steep (VS)		
DD	Drainage density (km km^−2^)	Topographical map	DD

### Fish Species Data

We collated data on fishes of the Himalayan rivers from published sources, documents, checklists and augmented this with primary data from our regular field surveys undertaken during the last six to eight years (see Appendix S1). We also used online sources (www.fishbase.org) for supplementing data on diversity and distributions of the Himalayan fishes. The elevational distribution ranges were available for only 179 (60%) out of reported 298 species, which form the basis of our analyses (however, nomenclatural changes indicate that there may be marginally less than 298) (Table S1). Fish species of Teesta river were assembled from a number of sources (see Appendix S1). Teesta is one of the major tributaries of Brahmaputra river system and constitutes the main drainage channel of Sikkim in the Eastern Himalaya. The river originates from Teesta Khangse glacier in the North Sikkim at 6280 m elevation; the study area lies between 27°04′43′′–27°59′57′′ N and 88°26′09′′–88°49′48′′ E falling within the political boundaries of Sikkim, India (Figure 1).

**Figure 2:**
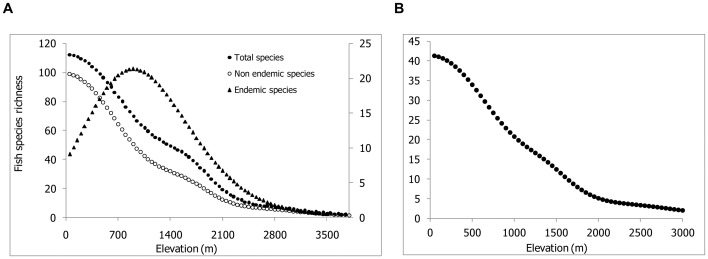
Fish species richness plots along the elevational gradient in the Himalaya. (A) total species richness (n = 179), non endemic species richness (n = 150) and endemic species richness (n = 29). (B) Species richness plots along the elevational gradient in the Teesta river. The fitted lines for total richness, non endemic richness in the Himalayan rivers and total fish species richness in the Teesta river represent a GAM model. However, for the endemic species of the Himalayan rivers the fitted line represents GLM model.

**Table 2:** Summary of the regression models between fish species richness and the Himalayan elevational gradient.

Categories	Null deviance	GLM^a^	GLM residual deviance	GAM^b^	GAM residual deviance
Total fishes	2732.394	2 (<0.001)	91.426	5 (<0.001)	48.043
Endemic fishes	478.4367	3 (<0.01)	35.632	n.s. (>0.05)	−
Non endemic fishes	2322.981	2 (<0.05)	73.496	5 (<0.001)	47.064
Total Teesta river fishes	667.247	2 (<0.001)	16.351	5 (<0.001)	7.888

a For GLM the respective best-fit polynomial order refers to a test against no relationship and with each other.

b For GAM the respective degrees of freedom are given and refer to a test against the given GLM model.

n.s. - not significant.

### Environmental Variables Data

The various environmental variables considered for this study were divided into four categories, viz. physico-chemical, biological, physiographic and topographic (Table 1). These variables were selected after removal of those that showed collinearity between them, based on variance inflation factor (VIF) test (see numerical analyses below). Each of these environmental data sets and their analyses is briefly described below. All necessary permits were obtained for the described field studies. We obtained permission from all the relevant Departments of Government of Sikkim (Permit No. GOS/H-II/97/56(Part)/314 dated 1.06.2005 & Permit No. 31/P&S/GOS/FEWMD dated 27.07.2005).

#### Physico-chemical data set

The details of physico-chemical data used in the analysis are given in Table 1. Water sampling was carried out for two years consecutively (2005–2006) in the Teesta river. Samples were collected from 10 sites from the river along elevational gradient (50–3800 m) to cover tropical, sub-tropical, temperate and alpine eco-climatic zones (Figure 1). Three replicates of water samples were collected at each site during pre-monsoon (March-April), monsoon (July) and post-monsoon (October) seasons. We followed standard methods for collection and physico-chemical analyses of water samples for various parameters described in Table 1 [32]–[34]. Temperature, pH and turbidity of water samples were recorded with the help of a graduated mercury thermometer, pH scan (Eutech Instruments, Singapore) and Nephelometer (Digital Turbidity Meter, EI Products, India) respectively. We measured total dissolved solids (TDS) and conductivity of water samples with the help of TDScan 1and TDScan 3 (Eutech Instruments, Singapore ), respectively, while dissolved oxygen (DO) was measured with the help of an oxygen test kit (Aquamerck, Germany), which is based on Winkler’s iodometric method [35]. We calculated average, maximum, minimum, range and standard deviation for each variable for statistical analyses.

**Table 3:** Ranges and Pearson’s correlation coefficient value of different environmental model variables against elevation in Teesta river.

Variables used for themodels	Range	Correlationcoefficient (r)
**Physico-chemical set**		
Tu.avg	6.1–23.7	−0.905**
T.avg	9.3–18.6	−0.852**
TDS.avg	13.3–23.3	0.188
C.avg	23.3–33.3	0.226
pH.avg	7.2–7.6	−0.793*
DO.avg	7.8–9.4	−0.142
**Biological set**		
P.avg	349–2782	0.946**
B.avg	4029–9262	0.925**
M.avg	247–727	0.349
**Physiographic set**		
D.avg	30.3–290.7	−0.922**
V.avg	1.0–1.6	0.685
G	5.0–55.0	0.831**
**Topographic set**		
A	700–5394.5	−0.976**
S	n.a	n.a.
DD	1.0–2.0	−0.809**

n.a. - not applicable.

** P<0.01;

* P<0.05.

#### Biological data set

Biological data set included estimation of densities of phytoplankton (suspended algae), phytobenthos and macro-invertebrates of Teesta river (Table 1). Samples in three replicates were collected from the same sites and in the same seasons as described in the case of physical and chemical parameters. We followed standard methods for collection and analyses of biological attributes for the riverine communities mentioned in Table 1 [32], [36].

For the collection of phytoplankton samples, 50 liters of water at each sampling site were filtered using plankton net made up of fine silk cloth (mesh size 25 µm). The residues were transferred to sampling vials and distilled water was added to these so that the total volume was made up to 100 ml. The samples thus obtained was preserved in Lugol’s solution and brought to the laboratory for further analysis. Each sample was thoroughly mixed and 1 ml from the sample was transferred to a Sedgewick-Rafter cell (SR cell) for analysis. Phytoplankton individuals were counted randomly in 100 chambers of the SR cell. The density of phytoplankton was estimated by the following equation:

where A is the average number of individuals per chamber; B, volume of the sample (ml); and L, the total volume of filtered water (liter).

Epilithic phytobenthos were sampled by scraping submerged surfaces of stones and boulders (substrate, measuring 3 cm^2^ area) with the help of a hard brush. The scrapings were transferred to sampling vials and distilled water was added to these so that the total volume was made up to 100 ml. The samples thus obtained were preserved in Lugol’s solution and brought to the laboratory for further analysis. Each sample was thoroughly mixed and 1 ml from the sample was transferred to a Sedgewick-Rafter cell (SR cell) for analysis. Phytobenthos were counted randomly in 100 chambers of SR cell. The density of phytobenthos was computed as follows (see [36]):

where N is the number of individuals counted; At, the total area (cm^2^) of chambers of SR cell; Vt, total volume (ml) of the sample; Ac, the area (cm^2^) of total chambers of SR cell counted; Vs, volume of the analyzed sample (ml) in SR cell; and As, the surface area of the substrate scrapped.

**Table 4:** Summary statistics for the selection of model variables.

Name of the variable	AIC	Residual deviance	D^2^	Percentage change in D^2^
**Physico-chemical model**				
T.avg	67.07	19.29	0.783	–
T.avg+DO.avg	62.77	12.99	0.854	9.07
T.avg+DO.avg+C.avg	57.26	5.474	0.938	9.84
Stepwise regression (AIC; backward elimination & forward selection)	56.00	4.224	0.952	1.49
All variables	62.36	4.577	0.948	–
**Biological model**				
P.avg	59.33	11.55	0.870	–
P.avg+B.avg	56.23	6.447	0.927	6.55
Stepwise regression (AIC; backward elimination & forward selection)	56.21	2.425	0.973	4.96
All variables	57.02	5.24	0.941	–
**Physiographic model**				
D.avg	66.37	18.58	0.791	–
Stepwise regression (AIC; backward elimination & forward selection)	50.98	1.202	0.986	24.65
All variables	66.58	16.80	0.811	–
**Topographic model**				
A	55.53	7.745	0.913	–
A+DD	54.77	4.992	0.944	3.40
Stepwise regression (AIC; backward elimination & forward selection)	51.24	1.459	0.984	4.24
All variables	58.34	4.563	0.949	–
**Synthetic model**				
A	55.53	7.745	0.913	–
A+D.avg	54.30	4.522	0.949	3.94
Stepwise regression (AIC; backward elimination & forward selection)	50.98	1.202	0.986	3.90
All variables	60.55	0.771	0.991	–

Null deviance = 88.77; d.f. = 8.

Macro-invertebrates attached to the substrate (mainly stones) were collected at random in the net of a square-foot Surber’s sampler. The substrate was disturbed and stirred thoroughly in order to dislodge all the attached macro-invertebrates. The individuals retained in the net were collected in a sampling vial. Samples were preserved in 70% alcohol and brought to the laboratory for further analysis. The macro-invertebrates were counted after identifying them under a compound microscope up to family level [37]. The density was calculated as number of individuals per unit sampling area.

We calculated average, maximum, minimum, range and standard deviation of densities for each of the taxonomic groups sampled and analyzed.

#### Physiographic data set

Physiographic data set included estimating water discharge, water current velocity and river gradient (Table 1). Water discharge was estimated by velocity–area method [38]. Width and average depth of river at a sampling location were measured directly, while water current velocity was measured using float method (see [34]). Average, maximum, minimum, range and standard deviation values were calculated for water discharge and water current velocity. Riverbed gradient at various sampling sites was calculated using Hack’s gradient index [39], [40] with the help of topographic map of the basin (see details below).

#### Topographic data set

Topographic data set included surface area of the drainage basin (basin area), slope, and drainage density (Table 1). A base map of the study area covering the basin was prepared to obtain topographic data set. River basin boundary, main river channel and its tributaries (drainage) were digitized from Survey of India (SOI) topographic sheets at 1∶50,000 scale using Arc GIS ver. 9.1 software. We calculated basin area of the river and its tributaries at each sampling site, following watershed boundaries, from the base map by simple querying using ArcGIS ver. 9.1. We used contour data of digitized SOI topographic sheets to generate Triangulated Irregular Network (TIN) model and grid raster model using ArcGIS ver. 9.1 to prepare a slope map of the river basin. The slopes of the river basin were classified into seven categories (see [41]) with minor modifications. The following slope categories were identified: (i) escarpment (>70%), (ii) very steep (50–70%), (iii) steep (30–50%), (iv) moderately steep (15–30%), (v) strongly sloping (8–15%), (vi) moderately sloping (2–8%), and (vi) gently sloping (up to 2%). Drainage density of the basin, upstream of each sampling point, was estimated by calculating total length of first- to seventh-order river channels using distance measuring tool of ArcGIS 9.1 and dividing the total length of the river channels by the basin area up to that sampling point.

**Figure 3:**
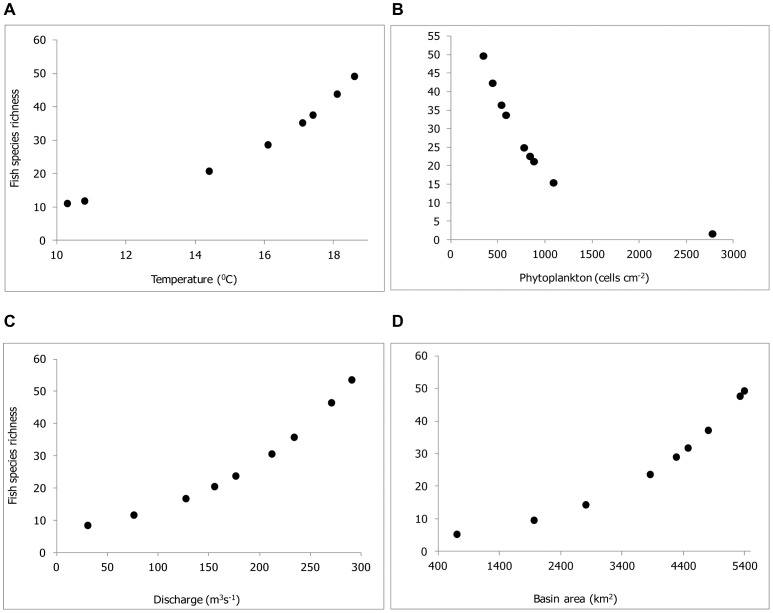
The significant variables, which effected maximum changes in percentage D^2^ values. (A) Water temperature represents physico-chemical model. (B) Phytoplankton density represents biological model. (C) Water discharge represents the physiographic model. (D) Basin area represents the topographic model. Discharge was the most important determining factor of fish species richness pattern followed by basin area and water temperature in decreasing order.

**Table 5:** Summary statistics of linear (l) and quadratic (q) parameters of variables for selected models and resultant effects of parameter removal on model performance.

Variable	Linear parameter (*l*)			Quadratic parameter (*q*)			Percentage change in D^2^		
	Estimate	SE	P-value	Estimate	SE	P-value	*−l*	*−q*	*−* (*l+q*)
**Physico-chemical model**									
(Intercept)	–	–	–	−4.328	1.801	*	–	–	–
T.avg	n.a.	n.a.	n.s.	0.009	0.001	***	n.a.	89.91	n.a.
DO.avg	n.a.	n.a.	n.s.	0.052	0.014	***	n.a.	17.69	n.a.
C.avg	n.a.	n.a.	n.s.	0.002	0.001	*	n.a.	7.38	n.a.
**Biological model**									
(Intercept)	5.390E-01	2.553E+00	n.s.	–	–	–	–	–	–
P.avg	−1.321E-03	5.549E-04	*	4.763E-07	2.981E-07	†	6.13	2.70	8.83
B.avg	1.637E-03	9.598E-04	†	−1.759E-07	9.179E-08	†	3.37	4.26	7.63
**Physiographic model**									
(Intercept)	1.303E-01	5.667E-01	n.s.	–	–	–	–	–	–
D.avg	2.852E-02	5.878E-03	***	−5.589E-05	1.464E-05	***	36.19	19.59	n.a.
**Topographic model**									
(Intercept)	−1.264E-01	7.337E-01	n.s.	–	–	–	–	–	–
A	1.349E-03	3.854E-04	***	−1.166E-07	4.955E-08	*	18.1	7.08	n.a.
DD	n.a.	n.a.	n.s.	n.a.	n.a.	n.s.	n.a.	n.a.	n.a.
**Synthetic model**									
(Intercept)	1.303E-01	5.667E-01	n.s.	–	–	–	–	–	–
D.avg	2.852E-02	5.878E-03	***	−5.589E-05	1.464E-05	***	36.19	19.59	n.a.
A	n.a.	n.a.	n.s.	n.a.	n.a.	n.s.	n.a.	n.a.	n.a.

SE - Standard error; ***−*** *l* - removal of linear parameter; ***−*** *q* - removal of quadratic parameter; n.a. - not applicable;

n.s. - not significant.

*** P<0.001;

** P<0.01;

* P<0.05;

† P<0.1.

### Statistical Analyses

#### Species richness

We collated fish species richness of 16 Himalayan rivers along elevational gradient between 50 and 3800 m (see Table S1). The lowermost elevational point represents the southernmost boundary of the Himalaya, while upper elevation marks the limit of fish distribution in the Himalayan rivers. The Himalayan elevational gradient under investigation was divided into 76 equal 50 m vertical bands assuming that no species has elevational distribution range of less than 50 m. This represents an estimate of gamma diversity of fishes, defined as total richness of an entire horizontal elevational band (see [14]). We plotted total, non-endemic and endemic fish species richness against the elevational gradient using published data on distribution and elevational ranges of fishes found in all the Himalayan rivers (see Appendix S1) to interpolate species’ presence between maximum and minimum elevations.

In order to test the drivers of the observed species richness patterns, an empirical study was carried out in the Teesta river along elevational gradient between 50 and 3000 m. As in case of the Himalayan rivers, 50 m elevation represents the lowermost limit and 3000 m the uppermost limit beyond which no fishes have been reported in the Teesta river. We divided the Teesta river elevational gradient into 60 vertical bands of 50 m each. We followed interpolation method to estimate the number of fish species in each elevation band, which represents species richness at that band (see [6]). We assumed that a species is represented at all the elevations between its minimum and maximum elevational records.

The relationship between the species richness and elevation was assessed using generalized linear model (GLM; [42]) and generalized additive model (GAM; [43]). GLM with Poisson error distribution and logarithmic link with up to third-order polynomials was tested against no relationship between species richness and elevation; all polynomial orders were also tested against each other to find the most significant model. On the basis of minimum residual deviance value, we selected the best-fit GLM [44]. The best-fit GLM was then tested against the GAM for total, non-endemic and endemic fish species richness of the Himalayan rivers and species richness in the Teesta river using a cubic smoother spline with 5 degrees of freedom. GAM was chosen because it is a non-parametric regression, which allows data to determine shape of the response curve instead of being limited by shapes available in parametric regression [44]. The significance tests of all the polynomial orders were carried out using both chi-square and F-test due to over-dispersion of the models [44], [45]. The fitted lines in GAM plot do not imply any causality and are only meant to aid the reader’s eye.

#### Species richness drivers

We used GLM to analyze the relationship between species richness in Teesta river and various sets of environmental variables using Poisson error distribution and logarithmic link [46], [16]. We used secondary data for interpolating fish species richness since it was not logistically possible to sample all the environmental variables and fishes at the same sites and even if we could it would not have changed the result. Species richness at each elevational band served as the response variable in all the models. The model building in this study was largely based on a previous study [24] with some modifications. We developed separate models for each set of variables described earlier and in each model groups of highly correlated variables having variance inflation factor (VIF) of >10 were eliminated to avoid problem of collinearity.

In the first step the best variable, with least Akaike’s information criterion (AIC) [47] and maximum explained deviance (D^2^) was selected for further GLM analysis [48], [24]. In second step we assumed that a combination of variables could better predict species richness, therefore, the selected best variable from each environmental set was combined successively with other variables of the same set to achieve greater D^2^ and lower AIC values compared to the first step. We used all possible *n*-variable combinations to determine the best *n*-variable model [24]. Step-wise regression analysis using backward elimination and forward selection criterion, based on AIC, was carried out in the third step. In this step linear and quadratic terms of the selected combination of variables from second step were included. Stepwise regression analysis removed the non-significant variables (z-statistic) and ultimately yielded the best combination of significant variables and their terms with the lowest AIC and the highest D^2^ values as compared to their individual and/or linear combinations of the previous steps. The resultant variables from third step and their terms after stepwise regression analyses were selected for further modelling.

Finally, a synthetic model was generated using the combination of best performing variables, selected from each environmental variable set, after stepwise regression analyses. Using the best performing variables we repeated model-building exercise to obtain the best predictor of fish species richness pattern.

We calculated relative importance of variables for the model performance, by removing them separately and in combination from GLM models in order to characterize the models. The variables and their terms, which accounted for maximum lowering of percentage D^2^ were selected as the most important ones for the model. The strength of the model was evaluated with 9-fold cross-validations. For robustness of results, the mean of 90 internal cross-validations was used. All the statistical analyses were performed using R 2.14.0 software [49].

**Table 6:** Test of model robustness by cross-validation: D^2^ values represent model fits while MAE represent mean absolute errors in number of species for the five proposed models.

Model	Number of		D^2^		MAE	
	Variables	Parameters	D^2^	9-foldCV^*^	MAE	9-foldCV^*^
Physico-chemical	3	3	0.952	0.712	3.300	5.275
Biological	2	4	0.973	0.755	2.264	5.986
Physiographic	1	2	0.986	0.636	1.514	2.313
Topographic	1	2	0.984	0.850	1.460	2.057
Synthetic	1	2	0.986	0.636	1.514	2.313

CV*, mean of 90 internal cross-validations (9-fold).

## Results

### Species Richness

The total and non-endemic species richness in the Himalayan rivers showed monotonic decrease with increasing elevation (Figure 2A). Endemic species richness, however, showed a unimodal pattern along elevational gradient with a broad peak between 700–1500 m. The trend of decreasing fish species with increasing elevation recorded for the Himalayan rivers, in general, was also observed in the Teesta river (Figure 2B). We could not plot species richness pattern of endemic fishes in the Teesta due to a few endemics having been reported from this river. The patterns in species richness for the Himalayan rivers and the Teesta were consistent with Rapoport’s rule. GAM model explained total and non-endemic species richness patterns in the Himalayan rivers as well as in the Teesta significantly better than GLM model (P<0.001; Table 2).

### Drivers of Species Richness

Ranges of different environmental model variables and their Pearson’s correlation coefficient values for the Teesta are given in Table 3. In the physico-chemical data set we observed significant negative correlation of turbidity (r = −0.905; P<0.01), water temperature (r = −0.852; P<0.01) and pH (r = −0.793; P<0.05) with elevation in the Teesta. There was a significant positive correlation for phytoplankton (r = 0.946; P<0.01) and phytobenthos (r = 0.925; P<0.01) with elevation. In the physiographic data set, water discharge and river gradient showed significant negative and positive correlation (r = −0.922; P<0.01 and r = 0.831; P<0.01), respectively with elevation. In topographic data set, basin area and drainage density showed significant negative correlations (r = −0.976; P<0.01 and r = −0.809; P<0.01), respectively with elevation.

In the model building procedure the first selected variable was the one with the least AIC and the highest D^2^ values as compared to other variables in its respective data set (Table 4). The following selected variables were shown to be significant factors: average water temperature (AIC = 67.07; D^2^ = 0.783), average density of phytoplankton (AIC = 59.33; D^2^ = 0.870), average water discharge (AIC = 66.37; D^2^ = 0.791) and basin area (AIC = 55.53; D^2^ = 0.913) in physico-chemical, biological, physiographic and topographic models, respectively. Linear combination of various variables in physico-chemical, biological and topographic models served as better predictors of fish species richness since they were able to achieve lesser AIC and higher D^2^ values as compared to a single variable (Table 4).

In the physico-chemical model, we observed that linear combination of water temperature, dissolved oxygen and electrical conductivity had lower AIC value (57.26) and higher D^2^ value (0.938). Likewise, linear combination of phytoplankton and phytobenthos served as better predictors in the biological model (AIC = 56.23; D^2^ = 0.927). In the topographic model linear combination of basin area and drainage density was better predictor of fish species richness (AIC = 54.77; D^2^ = 0.944).

Stepwise regression analyses further improved each model by achieving the least AIC and the highest D^2^ values as compared to individual and/or linear combinations of various environmental variables (Table 4). In case of physico-chemical model, inclusion of quadratic terms of selected variables resulted in lesser AIC and higher D^2^ values, therefore, the final model included only the quadratic terms of water temperature, dissolved oxygen (DO) and electrical conductivity while excluding their linear terms (AIC = 56.00; D^2^ = 0.952). For the biological model, step-wise analyses revealed that the combination of linear and quadratic terms of density of phytoplankton and phytobenthos were better predictors of fish species richness as compared to their linear combination alone (AIC = 56.21; D^2^ = 0.973; Table 4). Water discharge and basin area were better predictors when their linear and quadratic terms were included together in the final physiographic and topographic models, respectively (AIC = 50.98; D^2^ = 0.986; AIC = 51.24; D^2^ = 0.984; Table 4). Even as all the models reported high D^2^ values (>0.90), after step-wise regression analyses the highest D^2^ value (0.986) was recorded for water discharge in the physiographic model (Table 4).

In the synthetic model, basin area was found to be the best driver of species richness with least AIC value (55.53) and the highest D^2^ value (0.913) among the best predictor combination of variables (Table 4). Nevertheless, the linear combination of basin area and water discharge yielded lower AIC (54.30) and higher D^2^ (0.949) values compared to the basin area alone. However, the final model included only the linear and quadratic terms of water discharge values as the most significant predictor of species richness (AIC = 50.98; D^2^ = 0.986; Table 4) as revealed by the step-wise regression analyses.

The relative importance and effect of the variable removals on model performance were estimated (Table 5). Water temperature was the most significant variable in physico-chemical model in its quadratic term in explaining fish species richness pattern (Figure 3A) as it changed D^2^ value of the model by 89.91% on its removal. For the biological model, species richness pattern was better related to the combination of linear and quadratic terms of phytoplankton (Figure 3B) since their removal resulted in 8.83% change in D^2^ as compared to 7.63% when phytobenthos were removed. In the physiographic model, the removal of linear term of water discharge lead to 36.19% change in D^2^ compared to 19.59% change when its quadratic term was excluded. Thus, species richness was better predicted by water discharge in its linear term (Figure 3C). Likewise, basin area was also more important in its linear term (change in D^2^ by 18.1%) compared to the quadratic term (change in D^2^ by 7.08%). Thus, species richness was better predicted by basin area in its linear term (Figure 3D).

All the five models were found to be quite robust after being subjected to a 9-fold cross-validation as they were able to achieve a minimum D^2^ value of 0.50 (Table 6). After cross-validations, D^2^ values of all the models ranged from 0.636 to 0.850; lower values were recorded in physiographic and synthetic models while higher values were recorded in the topographic model. The cross-validated mean absolute error (MAE) in species richness ranged between 5.986 (for biological model) and 2.057 (for topographic model). Thus, all the models were quite strong with low error of prediction in determining fish species richness patterns as suggested by cross validation analyses.

In the ultimate analysis synthetic and physiographic models emerged as the best predictors of fish species richness in the Teesta (AIC = 50.98; D^2^ = 0.986) followed by topographic model (AIC = 51.24; D^2^ = 0.984), physico-chemical model (AIC = 56.00; D^2^ = 0.952) and biological model (AIC = 56.21; D^2^ = 0.973). Clearly, synthetic and physiographic models included water discharge in its linear and quadratic terms, it was considered to be the most important driver for fish species richness pattern.

## Discussion

### Species Richness Patterns

Total fish species richness and non-endemic richness in the Himalayan rivers showed a gradual decline with the increasing elevation, supporting Rapoport’s rule, but a mid domain effect was evident in the endemic fish species in this study. Our results are in contrast to a recent study on tree and bird elevational gradients of the Sikkim Himalaya where the authors reported that the species richness in these taxonomic groups peaked at intermediate elevations [27]–[28]. Our investigations are, however, in agreement with an earlier study on the fishes in the neighboring Himalayan Yangtze river basin, which reported that the diversity of the freshwater fishes decreased gradually with elevation, but endemic species distribution showed a unimodal peak [18]. These results indicate that richness gradients of taxonomic groups inhabiting terrestrial and aquatic ecosystems are likely to be different. Further, it is evident from our study that fish species richness patterns are influenced by various environmental variables like water discharge, basin area and temperature in that order of importance. More importantly, this study strengthens the equivocal debate that a uniform standard hypothesis explaining species richness across spatial extents is unlikely.

We show that the majority of the Himalayan fish endemics (58.6%) are clustered between 700 and 1500 m. These results, therefore, support the conclusion that endemic species peak towards the middle of an elevational gradient [4], [14]. Notwithstanding the absence of mid domain effect reported in this study, it is likely that the observed trends of fish species gradients could be linked to low proportion (9%) of endemics as compared those in the rivers of southern hill region of Western Ghats (40.9%) in India and the neighboring northern Yangtze river (49.1%) in China [50], [18]. The low fish endemism in the Himalayan rivers is possibly a result of lack of climatic and geographic isolation of these rivers - phenomena that are known to enhance endemism [15]. We also show that the maxima of the total species richness and the endemic species richness do not overlap as has been reported for other taxonomic groups in the Himalaya [6]. However, the overlapping patterns reported by these authors were for plant species, therefore, the comparisons may not be valid. Irrespective of the taxonomic and ecosystem differences, the varying patterns of total and endemic species richness in the fresh water ecosystems raise an important question about the validity of generalized theories in macroecology. This study provides support to elevational Rapoport effect [3] as opposed to mid-domain effect as far as total and non-endemic richness patterns are concerned, but agrees, albeit in a limited sense, with mid domain effect in so far as endemics are concerned [14].

### Fish Species Diversity Drivers

Various environmental, geographic and topographic features are often described as determinants of species richness patterns along elevational gradients [51]. How these factors influence the diversity and distribution of species has until recently been debated equivocally [52]. In order to determine the drivers of fish species richness in the Himalaya, we studied influence of various environmental factors on the fish species richness in the Teesta river. There was similarity in fish species richness patterns between the Himalayan rivers collectively and the Teesta specifically. Fish species richness decreased with increasing elevation in both cases due to a proportional relationship between local and regional richness (see [53]).

Our studies indicate that various environmental factors influence distribution of fish species richness differently and the relationship varies in magnitude. Generalized linear model identified water discharge, basin area, temperature and phytoplankton as the most important factors influencing species richness. However, the synthetic model, which represents close to the natural ecosystem state, showed a strong linear relationship of species richness with water discharge and basin area, in that order. This study, therefore, adds a new dimension to current macroecological theories dealing with drivers of species richness. Even as there is some support for species area theory, as indicated by the influence of basin area on fish species richness in the Himalayan rivers (see [18]) and also reported for other taxa in the terrestrial and aquatic ecosystems [9], [54], this study clearly shows that water discharge is the most significant factor driving fish species richness in the Himalayan rivers. Clearly, both water discharge and basin area increase towards downstream of a basin, therefore, the two are positively correlated. The natural corollary is that greater basin area must result in greater fish species richness [17]. Our investigations show that as the water discharge in the Teesta swells towards the lower elevations, fish species richness increases correspondingly, but support for species area theory (considering rive basin as area) is only second to river water discharge. As such empirical evidence to the view presented here is limited at present, but this study clearly shows that in comparison to water discharge the contribution of basin area to fish species diversity is less [55]. That said, higher water discharge could also be considered a surrogate measure of higher area resulting in greater habitat diversity in the rivers. In that sense species area theory could accordingly be modified in the case of aquatic ecosystems as species water discharge theory (see [56]).

Besides the size of river (a function more of water discharge than basin area due to role of precipitation), water temperature plays an important role in influencing fish diversity in the fresh water rivers. The Himalayan rivers at lower elevations are marked by slight annual fluctuations in water temperature, therefore, are inhabited by species assemblages with low physiological tolerance ranges (see [3]). In combination with high water flows, the lower elevations provide a wide range of available resources and niches to be occupied and exploited by large set of species (see [55]) resulting in higher fish species assemblages.

The low predictive performance of GLM models for species richness patterns with respect to other parameters of water chemistry (turbidity, TDS, Electrical Conductivity, pH, dissolved oxygen), biological (phytobenthos, macro-invertebrates), physiographic (water current velocity and gradient) and topography (slope, drainage density), confirm that these factors do not influence or at best contribute only a little to fish species richness in the Himalayan rivers.

Even though this study concerns essentially with diversity and distribution of fish species and drivers of their richness patterns, it potentially posits an important challenge to the unprecedented river regulation for hydropower generation in the Himalayan basins (see [21], [22], Grumbine RE and Pandit MK, unpublished results). Our results assume more significance because of the on-going large-scale hydropower development in the Himalaya with nearly 300 dams being built across these rivers. The river regulation activity would result in significant reduction of water discharge and alteration of natural diurnal flows, habitat fragmentation and barriers to fish migration [19]. The cumulative effects of water withdrawal are known to reduce freshwater biodiversity and lead to extinction of fish [57]. Worryingly, the zones of high species richness and endemism are also the sites of concentrated dam building and river regulation in the Himalaya [22].

To sum up, we found a greater applicability of Rapoport’s elevational rule in explaining the fish species richness pattern for the Himalayan rivers. Water discharge emerged as the best predictor for fish species richness pattern among 15 different environmental variables in the case study of Teesta river. The results of this study strongly advocate that the drivers of richness gradients in terrestrial and aquatic ecosystems are likely to be different. We recommend greater care to be exercised in design and execution of the ongoing dams being constructed across the Himalayan rivers.

## Supporting Information

Table S1 Fish fauna of Himalayan rivers and their distribution and conservation status.
(DOC)

Appendix S1 Detailed sources of data.
(DOC)
