# Correction: Elevational Gradients in Fish Diversity in the Himalaya: Water Discharge Is the Key Driver of Distribution Patterns

**DOI:** 10.1371/annotation/c24aa49a-7eb5-4612-bde3-8285b21921a0

**Published:** 2013-04-23

**Authors:** Jay P. Bhatt, Kumar Manish, Maharaj K. Pandit

In the Materials and Methods section, under the heading **Biological data set**, two equations are missing. The corrected text can be viewed here: http://plosone.org/corrections/pone.0046237.e001.cn.tif

