# Supplementary material for: Elevational Gradients in Fish Diversity in the Himalaya: Water Discharge Is the Key Driver of Distribution Patterns
Source: PLoS One. 2012 Sep 27;7(9):e46237. doi: 10.1371/journal.pone.0046237 (PMC3459831; doi:10.1371/journal.pone.0046237)
Supplement: Table S1 — Fish fauna of Himalayan rivers and their distribution and conservation status. (DOC) [file pone.0046237.s001.doc]

**Table S1. Fish fauna* of the Himalayan rivers and their distribution and conservation status.**

| **S.No.** | **Family/Species** | **Distribution** | **Status** | **Elevational Range (m)** |
| --- | --- | --- | --- | --- |
|  |  |  |  |  |
|  | **Notopteridae** |  |  |  |
| 1 | *Chitala chitala* (Hamilton 1822) | Indus, Ganga |  | - |
| 2 | *Notopterus notopterus* (Pallas 1769) | Ganga, Brahamputra |  | up to 120 |
|  | **Anguillidae** |  |  |  |
| 3 | *Anguilla bengalensis* (Gray 1831) | Ganga, Brahamputra |  | 50 - 1450 |
|  | **Clupeidae** |  |  |  |
| 4 | *Gudusia chapra* (Hamilton 1822) | Ganga |  | up to 1500 |
| 5 | *G. variegata* (Day 1870) | Ganga |  | up to 1500 |
|  | **Engraulidae** |  |  |  |
| 6 | *Setipinna phasa* (Hamilton 1822) | Ganga | Endemic | 700 – 1500 |
|  | **Cyprinidae** |  |  |  |
| 7 | *Amblypharyngodon mola* (Hamilton 1822) | Indus, Ganga, Brahamputra |  | up to 600 |
| 8 | *Aspidoparia jaya* (Hamilton 1822) | Ganga, Brahamputra |  | up to 600 |
| 9 | *Bangana almorae* (Chaudhuri 1912) | Indus, Ganga |  | - |
| 10 | *B. dero* (Hamilton 1822) | Indus, Ganga, Brahamputra |  | up to 1600 |
| 11 | *B. diplostoma* (Heckel 1838) | Indus |  | - |
| 12 | *Barilius barila* (Hamilton 1822) | Indus, Ganga, Brahamputra |  | up to 600 |
| 13 | *B. bendelisis* (Hamilton 1807) | Indus, Ganga, Brahamputra |  | up to 600 |
| 14 | *B. dogarsinghi* Hora 1921 | Brahamputra | Endemic | up to 600 |
| 15 | *B. modestus* Day 1872 | Indus |  | 50 – 300 |
| 16 | *B. radiolatus* Günther 1868 | Ganga |  | - |
| 17 | *B. shacra* (Hamilton 1822) | Indus, Ganga, Brahamputra |  | 50 – 300 |
| 18 | *B. tileo* (Hamilton 1822) | Ganga, Brahamputra |  | 50 – 300 |
| 19 | *B. vagra* (Hamilton 1822) | Indus, Ganga, Brahamputra |  | 50 – 300 |
| 20 | *Bengala elanga* (Hamilton 1822)  = *Rasbora elanga* (Hamilton 1822) | Brahamputra |  | - |
| 21 | *Cabdio morar* (Hamilton 1822) | Indus, Ganga, Brahamputra |  | up to 500 |
| 22 | *Carassius auratus* (Linnaeus 1758) | Indus | Exotic | up to 1500 |
| 23 | *C. carassius* (Linnaeus 1758) | Indus, Ganga | Exotic | 500 – 2500 |
| 24 | *Catla catla* (Hamilton 1822) | Indus, Ganga, Brahamputra |  | up to 1200 |
| 25 | *Chagunius chagunio* (Hamilton 1822) | Indus, Ganga, Brahamputra |  | up to 350 |
| 26 | *Chela cachius* (Hamilton 1822) | Ganga |  | - |
| 27 | *C. cachius* pakistanicus Mirza | Indus |  | - |
| 28 | *Cirrhinus cirrhosus* (Bloch 1795) | Brahamputra |  | up to 500 |
| 29 | *C. mrigala* (Hamilton 1822) | Ganga, Brahamputra |  | up to 350 |
| 30 | *C. reba* (Hamilton 1822) | Ganga, Brahamputra |  | up to 350 |
| 31 | *Crossocheilus diplochilus* (Heckel 1838) | Indus, Ganga |  | up to 600 |
| 32 | *C. latius* (Hamilton 1822) = *C. sada* (Günther 1868) | Indus |  | up to 200 |
| 33 | *C. latius latius* (Hamilton 1822) | Indus, Ganga, Brahamputra |  | up to 600 |
| 34 | *Ctenopharyngodon idella* (Valenciennes 1844) | Indus, Ganga, Brahamputra | Exotic | - |
| 35 | *Cyclocheilichthys apogon* (Valenciennes 1842) | Ganga |  | - |
| 36 | *Cyprinion semiplotum*(McClelland 1839) | Ganga, Brahamputra |  | up to 400 |
| 37 | *Cyprinus carpio* *communis* Linnaeus 1758 | Indus, Ganga, Brahamputra | Exotic | - |
| 38 | *C. carpio* *specularis* Linnaeus 1758 | Indus, Ganga, Brahamputra | Exotic | - |
| 39 | *Danio dangila* (Hamilton 1822) | Ganga, Brahamputra |  | up to 498 |
| 40 | *D. rerio* (Hamilton 1822) | Indus, Ganga, Brahamputra |  | - |
| 41 | *Devario aequipinnatus* (McClelland 1839) | Indus, Ganga, Brahamputra |  | up to 700 |
| 42 | *D. devario* (Hamilton 1822) | Indus, Ganga, Brahamputra |  | up to 500 |
| 43 | *D. horai* (Barman 1983) | Brahamputra |  | up to 499 |
| 44 | *Diptychus maculatus*Steindachner 1866 | Indus, Ganga |  | 1500 - 3800 |
| 45 | *Esomus danricus* (Hamilton 1822) | Indus, Ganga, Brahamputra |  | up to 600 |
| 46 | *Euchiloglanis kishinouyei* Kimura 1934 | Ganga |  | - |
| 47 | *Exostoma labiatum*(McClelland 1842)  = *Schizothoraicthys labiatus* (McClelland 1842) | Indus, Ganga |  | - |
| 48 | *Garra annandalei* Hora 1921 | Ganga, Brahamputra |  | up to 1800 |
| 49 | *G. gotyla* (Gray 1830) | Indus, Ganga, Brahamputra |  | up to 600 |
| 50 | *G. gravelyi* (Annandale 1919) | Brahamputra |  | - |
| 51 | *G. kempi* Hora 1921 | Brahamputra |  | up to 900 |
| 52 | *G. lamta* (Hamilton 1822) | Indus, Ganga, Brahamputra |  | 300 - 3000 |
| 53 | *G. lamta* (Hamilton 1822)  = *G. prashadi* (Hora 1921) | Indus |  | - |
| 54 | *G. lissorhynchus* (McClelland 1842) | Ganga, Brahamputra |  | up to 900 |
| 55 | *G. mullya* (Sykes 1839) | Ganga |  | - |
| 56 | *G. naganensis* Hora 1921  = *G. tirapensis* (Datta & Barman 1984) | Brahamputra |  | - |
| 57 | *G. naganensis* Hora 1921 | Brahamputra |  | up to 400 |
| 58 | *G. nasuta* (McClelland 1838) | Ganga, Brahamputra |  | up to 600 |
| 59 | *G. rupecula* (McClelland 1839) | Ganga, Brahamputra |  | - |
| 60 | *G. gotyla stenorhynchus* Jerdon 1849 | Brahamputra |  | - |
| 61 | *Hypophthalmichthys molitrix* (Valenciennes 1844) | Indus, Ganga, Brahamputra | Exotic | - |
| 62 | *H. nobilis* (Richardson 1845) | Ganga | Exotic | - |
| 63 | *H. nobilis* (Richardson 1845) = *Aristichthys nobilis* (Richardson 1845) | Brahamputra | Exotic | up to 600 |
| 64 | *Labeo angra* (Hamilton 1822) | Ganga |  | up to 1650 |
| 65 | *L. bata* (Hamilton 1822) | Indus, Ganga, Brahamputra |  | up to 853 |
| 66 | *L. boga* (Hamilton 1822) | Indus, Ganga, Brahamputra |  | up to 849 |
| 67 | *L. caeruleus* Day 1877 | Ganga |  | - |
| 68 | *L. calbasu* (Hamilton 1822) | Indus, Ganga, Brahamputra |  | up to 350 |
| 69 | *L. dyocheilus* (McClelland 1839) | Indus, Ganga, Brahamputra |  | up to 851 |
| 70 | *L. fimbriatus* (Bloch 1795) | Ganga |  | up to 350 |
| 71 | *L. gonius* (Hamilton 1822) | Indus, Ganga, Brahamputra |  | up to 351 |
| 72 | *L. pangusia* (Hamilton 1822) | Indus, Ganga, Brahamputra |  | up to 400 |
| 73 | *L. rohita*(Hamilton 1822) | Indus, Ganga, Brahamputra |  | up to 850 |
| 74 | *Laubuca laubuca* (Hamilton 1822) | Ganga, Brahamputra |  | up to 400 |
| 75 | *Neolissochilus hexagonolepis* (McClelland 1839) | Ganga, Brahamputra |  | up to 1200 |
| 76 | *N. dukai* (Day 1878) | Brahamputra |  | - |
| 77 | *N. spinulosus* (McClelland 1845) | Ganga, Brahamputra | Endemic | up to 800 |
| 78 | *N. stevensonii* (Day 1870) | Brahamputra |  | up to 900 |
| 79 | *Opsarius barna* (Hamilton 1822) | Indus, Ganga, Brahamputra |  | up to 600 |
| 80 | *O. barna* (Hamilton 1822)  = *Barilius jayarami* (Barman 1985) | Brahamputra |  | - |
| 81 | *Oreichthys cosuatis* (Hamilton 1822) | Ganga |  | - |
| 82 | *Osteobrama cotio* (Hamilton 1822) | Ganga, Brahamputra |  | - |
| 83 | *O. neilli* (Day 1873) | Ganga | Endemic | up to 400 |
| 84 | *Pethia conchonius* (Hamilton 1822) | Indus, Ganga, Brahamputra |  | 300 - 1400 |
| 85 | *P. gelius* (Hamilton 1822) | Ganga |  | 300 - 1400 |
| 86 | *P. phutunio* (Hamilton 1822) | Indus, Ganga |  | up to 600 |
| 87 | *P. ticto* (Hamilton 1822) | Indus, Ganga, Brahamputra |  | 200 - 1500 |
| 88 | *Pseudolaguvia ribeiroi* (Hora 1921) | Ganga |  | - |
| 89 | *Puntius chola* (Hamilton 1822) | Indus, Ganga, Brahamputra |  | up to 600 |
| 90 | *P. chola* (Hamilton 1822)  = *P. tetrarupagus* (McClelland 1839) | Indus, Brahamputra |  | up to 600 |
| 91 | *P. clavatus* (McClelland 1845) | Ganga |  | - |
| 92 | *P. guganio* (Hamilton 1822) | Brahamputra |  | up to 900 |
| 93 | *P. punjabensis* (Day 1871) | Indus |  | 250 - 1500 |
| 94 | *P. sophore* (Hamilton 1822)  = *P. chrysopterus* (McClelland 1839) | Indus |  | up to 600 |
| 95 | *P. sophore* (Hamilton 1822) | Indus, Ganga, Brahamputra |  | up to 600 |
| 96 | *P. sophore* (Hamilton 1822)  = *P. stigma* (Cuvier and Valenciennes 1844) | Indus |  | up to 600 |
| 97 | *P. terio* (Hamilton 1822) | Indus, Ganga |  | up to 1000 |
| 98 | *P. vittatus* Day 1865 | Indus |  | - |
| 99 | *Raiamas bola* (Hamilton 1822) | Indus, Ganga, Brahamputra |  | up to 600 |
| 100 | *R. guttatus* (Day 1870)  = *Barilius guttatus* (Day 1870) | Ganga |  | - |
| 101 | *R. guttatus* (Day 1870) | Ganga |  | - |
| 102 | *Rasbora daniconius* (Hamilton 1822)  =*Parluciosoma daniconius* (Hamilton 1822) | Ganga |  | - |
| 103 | *R. daniconius* (Hamilton 1822) | Indus |  | 50 - 700 |
| 104 | *Salmophasia acinaces* (Valenciennes 1844) | Ganga |  | - |
| 105 | *S. bacaila* (Hamilton 1822) | Indus, Ganga, Brahamputra |  | - |
| 106 | *S. boopis* (Day 1874) | Brahamputra |  | up to 500 |
| 107 | *S. phulo* (Hamilton 1822) | Ganga, Brahamputra |  | - |
| 108 | *Schizopyge niger* (Heckel 1838) | Indus, Ganga | Endemic | 400 - 2000 |
| 109 | *Schizopygopsis stoliczkai*Steindachner 1866  = *Gymnocypris biswasi* Talwar 1977 | Indus | Endemic | 2000 - 3500 |
| 110 | *S. stoliczkai* Steindachner 1866 | Brahamputra |  | 1500 - 3000 |
| 111 | *Schizothoraichthys nepalensis* Terashima 1984 | Ganga (Rara lake) | Endemic | 2900 - 3000 |
| 112 | *S. planifrons* Heckel 1838 | Ganga |  | 400 - 2000 |
| 113 | *S. sinuatus* Heckel 1838 | Indus, Ganga, Brahamputra |  | 400 - 2000 |
| 114 | *Schizothorax curvifrons* (Heckel 1838) | Indus, Ganga |  | 1500 - 2000 |
| 115 | *S. curvifrons* (Heckel 1838)  = *Schizothoraichthys micropogon* (Heckel 1838) | Indus |  | 400 – 3000 |
| 116 | *S. curvifrons* (Heckel 1838)  = *S. longipinnis* (Heckel 1838) | Ganga | Endemic | 400 – 2000 |
| 117 | *S. esocinus* Heckel 1838 | Indus, Ganga | Endemic | 400 - 2000 |
| 118 | *S. esocinus* Heckel 1838  = *S. punctatus* Day 1877 | Indus |  | 400 – 2000 |
| 119 | *S. kumaonensis* Menon 1971 | Ganga | Endemic | 300 - 2300 |
| 120 | *S. labiatus* (McClelland 1842)  = *Racoma labiata* (McClelland 1842) | Indus |  | - |
| 121 | *S. labiatus* (McClelland 1842) | Indus, Ganga |  | 400 - 2000 |
| 122 | *S. macrophthalmus* Terashima 1984 | Ganga | Endemic | 400 - 2000 |
| 123 | *S. molesworthi* (Chaudhuri 1913) | Brahamputra |  | 400 - 2000 |
| 124 | *S. plagiostomus* Heckel 1838 | Indus, Ganga, Brahamputra |  | 300 - 2300 |
| 125 | *S. progastus* (McClelland 1839) | Indus, Ganga, Brahamputra | Endemic | 100 - 2300 |
| 126 | *S. raraensis* Terashima 1984 | Ganga (Rara lake) | Endemic | 2900 - 3000 |
| 127 | *S. richardsonii* (Gray 1832) | Indus, Ganga, Brahamputra | Endemic | 100 - 2300 |
| 128 | *Securicula gora* (Hamilton 1822) | Ganga |  | - |
| 129 | *Semiplotus modestus* Day 1870 | Brahamputra |  | up to 200 |
| 130 | *Systomus sarana* (Hamilton 1822) | Idus, Ganga |  | up to 600 |
| 131 | *Tor chelyinoides* (McClelland) | Indus, Ganga, Brahamputra |  | up to 1000 |
| 132 | *T. progeneius* (McClelland 1839) | Brahamputra |  | up to 1000 |
| 133 | *T. putitora* (Hamilton 1822) | Indus, Ganga, Brahamputra |  | up to 1000 |
| 134 | *T. putitora*(Hamilton 1822)  = *T. mosal* (Hamilton 1822) | Indus, Ganga |  | - |
| 135 | *T. tor* (Hamilton 1822) | Indus, Ganga, Brahamputra |  | up to 1000 |
|  | **Psilorhynchidae** |  |  |  |
| 136 | *Psilorhynchus balitora* (Hamilton 1822) | Ganga, Brahamputra |  | - |
| 137 | *P. homaloptera* Hora & Mukerji 1935 | Ganga, Brahamputra |  | - |
| 138 | *P. pseudecheneis* Menon & Datta 1964 | Ganga | Endemic | 900 - 1500 |
| 139 | *P. sucatio* (Hamilton 1822) | Ganga, Brahamputra |  | - |
|  | **Balitoridae** |  |  |  |
| 140 | *Aborichthys elongatus* Hora 1921 | Brahamputra | Endemic | - |
| 141 | *A. garoensis* Hora 1925 | Brahamputra |  | - |
| 142 | *A. kempi* Chaudhuri 1913 | Brahamputra |  | - |
| 143 | *A. tikaderi* Barman 1985 | Brahamputra |  | - |
| 144 | *Acanthocobitis botia* (Hamilton 1822) | Indus, Ganga, Brahamputra |  | up to 400 |
| 145 | *A. pavonaceus* (McClelland 1839) | Brahamputra |  | up to 200 |
| 146 | *A. rubidipinnis* (Blyth 1860) | Indus |  | - |
| 147 | *Balitora brucei* Gray 1830 | Indus, Ganga, Brahamputra |  | 300 - 1200 |
| 148 | *Bengala elanga* (Hamilton 1822) | Ganga |  | up to 400 |
| 149 | *Lepidocephalichthys arunachalensis* (Datta & Barman 1984) | Brahamputra | Endemic | up to 900 |
| 150 | *Nemacheilus carletoni* Fowler 1924 | Indus |  | 600 - 1500 |
| 151 | *N. corica*(Hamilton 1822) | Indus, Ganga, Brahamputra |  | - |
| 152 | *N. doonensis* Tilak & Husain 1977 | Ganga (Son) | Endemic | 300 - 500 |
| 153 | *N. drassensis* Tilak 1990 | Indus | Endemic | - |
| 154 | *N. nilgiriensis* Menon 1987 | Indus |  | - |
| 155 | *N. sikkimensis* Mani 1974 | Brahamputra |  | 1200 - 1800 |
| 156 | *Paraschistura montana* (McClelland 1838) | Indus, Brahamputra |  | 500 - 1800 |
| 157 | *Schistura alepidata* | Indus (Jhelum) |  | - |
| 158 | *S. beavani* (Günther 1868) | Indus, Ganga |  | 200 - 1000 |
| 159 | *S. devdevi* (Hora 1935) | Indus, Ganga, Brahamputra | Endemic | 100 - 900 |
| 160 | *S. himachalensis* (Menon 1987) | Ganga | Endemic | 500 - 1800 |
| 161 | *S. horai* (Menon 1952) | Indus |  | - |
| 162 | *S. kangjupkhulensis* (Hora 1921) | Brahamputra |  | - |
| 163 | *S. manipurensis* (Chaudhuri 1912) | Brahamputra |  | - |
| 164 | *S. multifasciata* (Day 1878) | Indus, Ganga, Brahamputra |  | 500 - 1800 |
| 165 | *S. prashadi* (Hora 1921) | Brahamputra |  | - |
| 166 | *S. punjabensis* (Hora 1923) | Indus |  | - |
| 167 | *S. rupecula* McClelland 1838 | Indus, Ganga, Brahamputra |  | - |
| 168 | *S. savona* (Hamilton 1822) | Indus, Ganga, Brahamputra |  | - |
| 169 | *S. scaturigina* McClelland 1839 | Indus, Ganga |  | up to 1000 |
| 170 | *S. zonata* McClelland 1839 | Ganga |  | - |
| 171 | *Triplophysa gracilis* (Day 1877) | Indus, Ganga |  | 1400 - 2500 |
| 172 | *T. kashmirensis* (Hora 1922) | Indus (Jhelum) | Endemic | 400 - 2500 |
| 173 | *T. ladacensis* (Günther 1868) | Indus |  | 2000 - 3500 |
| 174 | *T. marmorata* (Heckel 1838) | Indus | Endemic | 800 - 2000 |
| 175 | *T. stoliczkai* (Steindachner 1866) | Indus |  | 1000 - 3000 |
|  | **Cobitidae** |  |  |  |
| 176 | *Acantopsis choirorhynchos* (Bleeker 1854) | Brahamputra |  | - |
| 177 | *Botia almorhae* Gray 1831 | Indus, Ganga |  | 200 - 700 |
| 178 | *B. birdi* Chaudhuri 1909 | Indus |  | up to 600 |
| 179 | *B. dario* (Hamilton 1822) | Indus, Ganga, Brahamputra |  | up to 600 |
| 180 | *B.. dario* (Hamilton 1822)  = *B. geto* (Hamilton 1822) | Indus, Brahamputra |  | 500 – 1500 |
| 181 | *B. lohachata* Chaudhuri 1912 | Indus, Ganga |  | up to 900 |
| 182 | *B. rostrata* Günther 1868  = *B. dayi* Hora 1932 | Indus |  | - |
| 183 | *B. rostrata* Günther 1868 | Brahamputra |  | up to 900 |
| 184 | *Canthophrys gongota* (Hamilton 1822) | Ganga, Brahamputra |  | up to 600 |
| 185 | *Lepidocephalichthys guntea* (Hamilton 1822) | Indus, Ganga, Brahamputra |  | up to 500 |
| 186 | *Lepidocephalus nipalensis* (Shrestha) | Ganga (Kosi, Gandak) |  | - |
| 187 | *Pangio pangia* (Hamilton 1822) | Ganga, Brahamputra |  | up to 150 |
| 188 | *Syncrossus berdmorei* Blyth 1860 | Brahamputra |  | - |
|  | **Bagridae** |  |  |  |
| 189 | *Batasio batasio* (Hamilton 1822) | Ganga, Brahamputra |  | up to 800 |
| 190 | *B. tengana* (Hamilton 1822) | Brahamputra |  | - |
| 191 | *Hemibagrus menoda* (Hamilton 1822) | Indus, Ganga, Brahamputra |  | - |
| 192 | *Mystus bleekeri* (Day 1877) | Indus, Ganga, Brahamputra |  | - |
| 193 | *M. cavasius* (Hamilton 1822) | Ganga, Brahamputra |  | - |
| 194 | *M. montanus* (Jerdon 1849) | Brahamputra |  | - |
| 195 | *M. tengara*(Hamilton 1822) | Indus, Ganga |  | - |
| 196 | *M. vittatus* (Bloch 1794) | Indus, Ganga, Brahamputra |  | up to 500 |
| 197 | *Rita rita* (Hamilton 1822) | Indus, Ganga, Brahamputra |  | - |
| 198 | *Sperata aor* (Hamilton 1822) | Indus, Ganga |  | up to 200 |
| 199 | *S. seenghala* (Sykes 1839) | Indus, Ganga, Brahamputra |  | up to 250 |
|  | **Siluridae** |  |  |  |
| 200 | *Ompok bimaculatus* (Bloch 1794) | Indus, Ganga, Brahamputra |  | - |
| 201 | *O. pabda* (Hamilton 1822) | Indus, Ganga, Brahamputra |  | - |
| 202 | *O. pabo* (Hamilton 1822) | Brahamputra |  | - |
| 203 | *Pterocryptis indicus* (Datta, Barman & Jayaram 1987) | Brahamputra |  | - |
| 204 | *Wallago attu* (Bloch & Schneider 1801) | Indus, Ganga, Brahamputra |  | up to 300 |
|  | **Schilbeidae** |  |  |  |
| 205 | *Ailia coila* (Hamilton 1822) | Ganga, Brahamputra |  | 100 - 250 |
| 206 | *Clupisoma garua* (Hamilton 1822) | Ganga, Brahamputra |  | up to 1000 |
| 207 | *C. montana* Hora 1937 | Ganga |  | up to 1400 |
| 208 | *C. naziri* Mirza & Awan 1973 | Indus |  | - |
| 209 | *Eutropiichthys murius* (Hamilton 1822)  = *Pseudeutropius murius batarensis* (Shrestha 1980) | Ganga |  | - |
| 210 | *Neotropius atherinoides* (Bloch 1794) | Ganga, Brahamputra |  | - |
| 211 | *Silonia silondia* (Hamilton 1822) | Ganga |  | up to 300 |
|  | **Pangasiidae** |  |  |  |
| 212 | *Pangasius pangasius* (Hamilton 1822) | Ganga |  | - |
|  | **Amblycipitidae** |  |  |  |
| 213 | *Amblyceps mangois* (Hamilton 1822) | Indus, Ganga, Brahamputra |  | up to 600 |
|  | **Sisoridae** |  |  |  |
| 214 | *Badis badis* (Hamilton 1822) | Indus, Ganga, Brahamputra |  | up to 500 |
| 215 | *Bagarius bagarius* (Hamilton 1822) | Indus, Ganga, Brahamputra |  | 70 - 1450 |
| 216 | *B. yarrelli* (Sykes 1839) | Ganga |  | - |
| 217 | *Creteuchiloglanis kamengensis* (Jayaram 1966) | Brahamputra |  | - |
| 218 | *Erethistes pusillus* Müller & Troschel 1849 | Ganga |  | up to 600 |
| 219 | *Erethistoides montana* Hora 1950 | Brahamputra |  | up to 800 |
| 220 | *Eutropiichthys goongwaree* (Sykes 1839) | Ganga |  | - |
| 221 | *E. murius*(Hamilton 1822) | Ganga |  | - |
| 222 | *E. vacha* (Hamilton 1822) | Ganga |  | - |
| 223 | *Exostoma berdmorei* Blyth 1860 | Brahamputra |  | - |
| 224 | *E. labiatum* (McClelland 1842) | Ganga |  | - |
| 225 | *E. stuarti* (Hora 1923) | Brahamputra |  | - |
| 226 | *Gagata cenia* (Hamilton 1822) | Brahamputra |  | up to 500 |
| 227 | *G. gagata* (Hamilton 1822) | Ganga |  | - |
| 228 | *G. sexualis* Tilak 1970 | Ganga |  | 900 - 2000 |
| 229 | *Glossogobius giuris* (Hamilton 1822) | Indus, Ganga, Brahamputra |  | - |
| 230 | *Glyptosternon maculatum* (Regan 1905) | Indus, Ganga |  | - |
| 231 | *G. reticulatum* McClelland 1842 | Indus, Ganga |  | 1400 - 2000 |
| 232 | *Glyptothorax alaknandi* Tilak 1969 | Ganga (Alaknanda) | Endemic | 400 - 550 |
| 233 | *G. annandalei* Hora 1923 | Ganga, Brahamputra |  | 1250 - 1875 |
| 234 | *G. brevipinnis*Hora 1923 | Indus |  | up to 1100 |
| 235 | *G. cavia* (Hamilton 1822) | Indus, Ganga |  | 1250 - 1875 |
| 236 | *G. conirostris* (Steindachner 1867) | Indus, Ganga |  | 1250 - 1875 |
| 237 | *G. garhwali* Tilak 1969 | Ganga | Endemic | 400 - 1200 |
| 238 | G. gracilis (Günther 1864)  = *G. dakpathari* Tilak & Husain 1976 | Ganga (Yamuna) | Endemic | 300 – 500 |
| 239 | *G. gracilis* (Günther 1864) | Indus, Ganga |  | 1250 - 1880 |
| 240 | *G. indicus* Talwar 1991 | Ganga |  | 1250 - 1880 |
| 241 | *G. kashmirensis* Hora 1923 | Indus, Ganga | Endemic | 1250 - 1875 |
| 242 | *G. madraspatanus* (Day 1873) | Indus |  | - |
| 243 | *G. pectinopterus* (McClelland 1842) | Indus, Ganga |  | 1250 - 1875 |
| 244 | *G. punjabensis* Mirza & Kashmiri 1971 | Indus | Endemic | - |
| 245 | *G. sinensis* (Regan 1908) | Brahamputra |  | - |
| 246 | *G. stolickae* (Steindachner 1867) | Indus |  | 1250 - 1875 |
| 247 | *G. striatus* (McClelland 1842) | Brahamputra |  | up to 400 |
| 248 | *G. telchitta* (Hamilton 1822) | Indus, Ganga |  | 1250 - 1875 |
| 249 | *G. trilineatus* Blyth 1860 | Indus, Ganga, Brahamputra |  | 1250 - 1875 |
| 250 | *Gogangra viridescens* (Hamilton 1822)  = *Nangra punctata* (Day 1877) | Brahamputra |  | - |
| 251 | *G. viridescens* (Hamilton 1822) | Ganga, Brahamputra |  | - |
| 252 | *Hara hara* (Hamilton 1822) | Ganga |  | up to 1500 |
| 253 | *H. jerdoni* Day 1870 | Ganga |  | up to 900 |
| 254 | *Myersglanis blythii* (Day 1870) | Ganga | Endemic | 300 - 1500 |
| 255 | *Nangra nangra* (Hamilton 1822) | Ganga |  | - |
| 256 | *Parachiloglanis hodgarti* (Hora 1923) | Ganga, Brahamputra |  | - |
| 257 | *Pseudecheneis sulcata* (McClelland 1842) | Indus, Ganga, Brahamputra |  | - |
| 258 | *Pseudolaguvia ribeiroi* (Hora 1921)  = *Glyptothorax ribeiroi* Hora 1921 | Ganga,Brahamputra |  | 1250 – 1885 |
| 259 | *Sisor rabdophorus* Hamilton 1822 | Ganga, Brahamputra |  | - |
|  | **Clariidae** |  |  |  |
| 260 | *Clarias batrachus* (Linnaeus 1758) | Indus, Ganga, Brahamputra |  | up to 200 |
|  | **Heteropneustidae** |  |  |  |
| 261 | *Heteropneustes fossilis* (Bloch 1794) | Indus, Ganga, Brahamputra |  | up to 900 |
|  | **Chacidae** |  |  |  |
| 262 | *Chaca chaca* (Hamilton 1822) | Ganga |  | up to 900 |
|  | **Olyridae** |  |  |  |
| 263 | *Olyra horae* (Prashad & Mukerji 1929) | Brahamputra |  | - |
| 264 | *O. kempi* Chaudhuri 1912 | Brahamputra |  | - |
| 265 | *O. longicaudata* McClelland 1842 | Ganga |  | - |
|  | **Salmonidae** |  |  |  |
| 266 | *Oncorhynchus masou rhodurus* (Shaw 1796) | Indus, Ganga | Exotic | - |
| 267 | *O. mykiss* (Walbaum 1792) | Indus, Ganga | Exotic | - |
| 268 | *Salmo trutta fario* Linnaeus 1758 | Indus, Ganga, Brahamputra | Exotic | 1400 - 3800 |
| 269 | *S. trutta trutta* Linnaeus 1758 | Brahamputra | Exotic | - |
|  | **Belonidae** |  |  |  |
| 270 | *Xenentodon cancila* (Hamilton 1822) | Indus, Ganga, Brahamputra |  | up to 300 |
|  | **Aplocheilidae** |  |  |  |
| 271 | *Aplocheilus panchax* (Hamilton 1822) | Ganga |  | up to 900 |
|  | **Poeciliidae** |  |  |  |
| 272 | *Gambusia affinis* (Baird & Girard 1853) | Indus, Ganga, Brahamputra | Exotic | 500 - 1500 |
| 273 | *G. holbrooki* Girard 1859 | Indus | Exotic | 500 - 2000 |
|  | **Synbranchidae** |  |  |  |
| 274 | *Monopterus cuchia* (Hamilton 1822) | Ganga, Brahamputra |  | - |
|  | **Ambassidae** |  |  |  |
| 275 | *Parambassis baculis* (Hamilton 1822) | Indus, Ganga |  | - |
| 276 | *Pseudambassis ranga* (Hamilton 1822) | Ganga |  | - |
|  | **Chandidae** |  |  |  |
| 277 | *Chanda nama* Hamilton 1822 | Ganga, Brahamputra |  | up to 400 |
| 278 | *Pseudambassis ranga* (Hamilton 1822)  = *Chanda ranga* (Hamilton 1822) | Brahamputra |  | - |
|  | **Sciaenidae** |  |  |  |
| 279 | *Johnius coitor* (Hamilton 1822) | Brahamputra |  | up to 300 |
|  | **Nandidae** |  |  |  |
| 280 | *Nandus nandus* (Hamilton 1822) | Ganga, Brahamputra |  | up to 900 |
|  | **Cichlidae** |  |  |  |
| 281 | *Oreochromis mossambicus* (Peters 1852) | Ganga | Exotic | - |
| 282 | *O. niloticus* (Linnaeus 1758) | Ganga | Exotic | - |
|  | **Mugilidae** |  |  |  |
| 283 | *Rhinomugil corsula* (Hamilton 1822) | Ganga |  | up to 400 |
| 284 | *Sicamugil cascasia* (Hamilton 1822) | Ganga |  | - |
|  | **Anabantidae** |  |  |  |
| 285 | *Anabas testudineus* (Bloch 1792) | Ganga, Brahamputra |  | - |
|  | **Osphronemidae** |  |  |  |
| 286 | *Trichogaster fasciata* Bloch & Schneider 1801 | Indus, Ganga, Brahamputra |  | - |
| 287 | *T. lalius* (Hamilton 1822) | Ganga |  | - |
|  | **Channidae** |  |  |  |
| 288 | *Channa barca* (Hamilton 1822) | Brahamputra |  | - |
| 289 | *C. gachua* (Hamilton 1822) | Indus, Ganga, Brahamputra |  | up to 600 |
| 290 | *C. marulius*(Hamilton 1822) | Indus, Ganga, Brahamputra |  | up to 300 |
| 291 | *C. orientalis* Bloch & Schneider 1801 | Indus, Ganga, Brahamputra |  | up to 200 |
| 292 | *C. punctata* (Bloch 1793) | Indus, Ganga, Brahamputra |  | up to 300 |
| 293 | *C. stewartii* (Playfair 1867) | Ganga, Brahamputra |  | up to 300 |
| 294 | *C. striata* (Bloch 1793) | Indus, Ganga, Brahamputra |  | - |
|  | **Mastacembelidae** |  |  |  |
| 295 | *Macrognathus aral*(Bloch & Schneider 1801) | Ganga |  | - |
| 296 | *M. pancalus* Hamilton 1822 | Indus, Ganga, Brahamputra |  | - |
| 297 | *Mastacembelus armatus* (Lacepède 1800) | Indus, Ganga |  | up to 700 |
|  | **Tetraodonidae** |  |  |  |
| 298 | *Tetraodon cutcutia* Hamilton 1822 | Ganga |  | - |

*Fish taxonomic nomenclature followed according to Eschmeyer, W.N.(ed). Catalog of Fishes, California Academy of Sciences (<http://research.calacademy.org/research/ichthyology/catalog/fishcatmain.asp>).

- Data not available; Elevational distribution ranges are available for only 179 (60%) out of mentioned 298 species.

= Synonym
