# Supplementary material for: Elevational Gradients in Fish Diversity in the Himalaya: Water Discharge Is the Key Driver of Distribution Patterns
Source: PLoS One. 2012 Sep 27;7(9):e46237. doi: 10.1371/journal.pone.0046237 (PMC3459831; doi:10.1371/journal.pone.0046237)
Supplement: Appendix S1 — Detailed sources of data. (DOC) [file pone.0046237.s002.doc]

**Appendix S1.** Detailed sources of data.

1. Akhtar N (1991) The Northern Areas (Pakistan). Fisheries profile, feasible sites for trout culture and an overall sector development perspective. Report for Project PAK/91/008. Rome, FAO. 29p.
2. Akhtar N (1992) Pakistan's cold water fisheries and trout farming sector study: Trends, opportunities and challenges. Report for FAO/UNDP Projects PAK/88/048 and PAK/91/008. Rome, FAO. 75p.
3. Atkore VM (2005) Conservation status of fishes in the tributaries of Ramganga with special reference to golden mahseer (Tor putitora) Hamilton. Dissertation submitted to Saurashtra University, Rajkot, India.
4. CISMHE (2004) EIA/ EMP Studies of Kishanganga H.E. Project on Kishanganga river in Jammu & Kashmir. Report submitted to National Hydroelectric Power Corporation (NHPC) Ltd., Faridabad.
5. CISMHE (2005) EIA/ EMP Studies of Chamera H.E. Project (Stage-III) on Ravi river in Himachal Pradesh. Report submitted to National Hydroelectric Power Corporation (NHPC) Ltd., Faridabad.
6. CISMHE (2006) Carrying Capacity Study of Teesta Basin in Sikkim. Report submitted to Ministry of Environment & Forests Government of India, New Delhi.
7. CISMHE (2008) EIA/ EMP Studies for Drangdhuran-Dul (Pakal-Scheme) H.E. Project on Marusudar river in Kashmir, J&K. Report submitted to National Hydroelectric Power Corporation (NHPC) Ltd., Faridabad.
8. CISMHE (2008) EIA/ EMP Studies of Alaknanda H.E. Project on Alaknanda river in Uttaranchal. Report submitted to GMR Energy Ltd., Bangalore.
9. CISMHE (2008) EIA/ EMP Studies of Singoli Bhatwari H.E. Project on Mandakini river in Uttaranchal. Report submitted to Larson & Toubro Ltd., Chennai.
10. CISMHE (2009) EIA/EMP Studies of Demwe Lower H.E. Project on Lohit river in Arunachal Pradesh. Report submitted to Athena Demwe Power Pvt. Ltd., New Delhi.
11. CISMHE (2010) EIA/EMP Studies of Tawang Chhu Stage-I H.E. project on Tawang river in Arunachal Pradesh. Report submitted to National Hydroelectric Power Corporation (NHPC) Ltd., Faridabad.
12. CISMHE (2010) EIA/ EMP Studies of Tawang Chhu Stage-II H.E. project on Tawang river in Arunachal Pradesh. Report submitted to National Hydroelectric Power Corporation (NHPC) Ltd., Faridabad.
13. CISMHE (2011) EIA/ EMP Studies for Luhri H.E. Project on Sutlej river in Himachal Pradesh. Report submitted to Satluj Jal Vidyut Nigam Ltd., New Shimla, Himachal Pradesh.
14. CISMHE (2011) EIA/ EMP Studies of Lower-Siang H.E. project on Siang river in Arunachal Pradesh. Report submitted to Jaypee Associates, New Delhi.
15. CISMHE (2011) EIA/ EMP Studies of Hirong H.E. project on Siyom river in Arunachal Pradesh. Report submitted to Jaypee Associates, New Delhi.
16. Das RC, Mukherjee AB (2004) Final report for provision of fish way at the proposed Tista low dam project –IV dam structure of NHPC at Kalijhora. Report submitted to NHPC, Siliguri.
17. Khanna DR, Badola SP (1994) Foothill section of river Ganga – Habitat of adult mahseer. In: Nautiyal P (ed.) Mahseer the game fish. Dehradun: Jagdamba Prakashan. pp. B98-101.
18. Jhingran VG (1991) Fish and fisheries of India. Delhi: Hindustan Publishing Corporation.
19. **Kullander SO, Fang F, Delling B, Ahlander E (1999)** The fishes of the Kashmir Valley. In: Nyman L (ed) River Jhelum, Kashmir Valley: Impacts on the aquatic environment. Göteborg: Swedmar. pp. 99-167.
20. Mehta HS, Uniyal DP (2004) Pisces. Fauna of Western Himalaya (Part 2). Kolkata: Zoological Survey of India. pp. 255-268.
21. Menon AGK (1954) Fish geography of Himalayas. Proc. Natl. Sci. Acad. India 20: 467-93.
22. Menon AGK (1962) A distributional list of fishes of the Himalayas. J. Zool. Soc. India 14: 23-32.
23. Menon AGK (1999) Check list – Freshwater fishes of India. Rec. Zool. Surv. India. Paper No. 175: 1-366.
24. Petr T (1999) Coldwater fish and fisheries in Bhutan. FAO Fish. Tech. Pap. No. 385. pp. 6-12.
25. Sen, TK (2006) Pisces. Fauna of Arunachal Pradesh, State fauna Series. Zool. Surv. India 13: 317-396.
